# Novel Approaches in the Management of Mucormycosis

**DOI:** 10.1007/s12281-023-00463-3

**Published:** 2023-05-08

**Authors:** Frederic Lamoth

**Affiliations:** 1 Service of Infectious Diseases, Department of Medicine, Lausanne University Hospital, University of Lausanne, Rue du Bugnon 48, 1011 Lausanne, Switzerland; 2 Institute of Microbiology, Department of Laboratory Medicine and Pathology, Lausanne University Hospital, University of Lausanne, Rue du Bugnon 48, 1011 Lausanne, Switzerland

**Keywords:** Mucor, Rhizopus, Antifungal therapy, Fosmanogepix, Spore coat protein

## Abstract

**Purpose of Review:**

Invasive mucormycosis (IM), caused by fungi of the order Mucorales, is one of the deadliest fungal infection among hematologic cancer patients. Its incidence is also increasingly reported in immunocompetent individuals, notably with the COVID-19 pandemic. Therefore, there is an urgent need for novel diagnostic and therapeutic approaches of IM. This review discusses the current advances in this field.

**Recent Findings:**

Early diagnosis of IM is crucial and can be improved by Mucorales-specific PCR and development of lateral-flow immunoassays for specific antigen detection. The spore coat proteins (CotH) are essential for virulence of the Mucorales and may represent a target for novel antifungal therapies. Adjuvant therapies boosting the immune response, such as interferon-γ, anti-PDR1 or fungal-specific chimeric antigen receptor (CAR) T-cells, are also considered.

**Summary:**

The most promising perspectives for improved management of IM consist of a multilayered approach targeting both the pathogen and the host immune system.

## Introduction

Invasive mucormycosis (IM) is an invasive fungal disease due to filamentous fungi (molds) of the order Mucorales (e.g. *Mucor*, *Rhizopus*, *Lichtheimia*, *Rhizomucor* or *Cunninghamella*). These rapidly growing fungi have the ability to cause extensive tissue damages with inflammation and necrosis. Albeit relatively rare, IM is one of the deadliest infection among patients with hematologic cancer [1, 2, 3•, 4]. It may also affect patients with other immunosuppressive conditions, such as solid-organ transplant recipients and apparently immunocompetent individuals, such as those with uncontrolled diabetes mellitus and ketoacidosis [1, 2, 5, 6]. Recently, a high incidence of IM has been reported among patients with severe Coronavirus disease 2019 (COVID-19), in particular those with diabetes [7]. The SARS-CoV-2 virus may affect the pancreatic islets via the angiotensin-converting enzyme 2 (ACE2) receptor and decompensate pre-existing diabetes [8]. Moreover, insulin resistance can result from corticosteroids that are used for the treatment of COVID-19.

Pulmonary mucormycosis is the predominant presentation of IM among severely immunocompromised patients who may also present dissemination of the disease to other organs, while rhino- orbito-cerebral (ROC) mucormycosis is more frequently observed among diabetic patients [2]. Cutaneous mucormycosis mainly occurs after trauma or burns, in particular when wounds are contaminated by dust or mud (e.g. tornadoes, hurricanes, flooding) [9, 10].

IM represents the second most frequent invasive mold disease after invasive aspergillosis in immunocompromised patients accounting for 8–9% and 2–3% of cases among hematopoietic stem cell and solid organ transplant recipients, respectively [6, 11]. The incidence of IM greatly varies from one country to another with the highest incidence reported from South Asia (14 cases per 100,000 inhabitants in India and Pakistan) and lower incidences in Europe and USA (< 0.5 per 100,000) [12], which may be related to local climatic conditions, prevalence of diabetes and access to healthcare systems. Most epidemiological studies suggest that the incidence of IM is increasing, as the consequence of the expanding population of patients with prolonged immunosuppressive conditions in developed countries and the increasing prevalence of diabetes mellitus in low-income countries [12–16]. However, other triggers might be involved, such as improved diagnostic procedures (e.g. molecular methods) and possibly the impact of global warming and the increasing incidence of natural disasters. Notably, the COVID-19 pandemic has been associated with a huge rise of reported cases of IM, especially in India where its incidence has doubled [8, 17].

The mortality rate of IM remains high (about 40–50%) and the prognosis did not significantly improve over the last decades [3•, 18–20]. A similar poor outcome has been reported among patients with COVID-19–associated mucormycosis (CAM) [7, 8, 17]. A lower mortality rate has been reported among diabetic patients with ROC compared to that in hematologic patients with other localizations of the disease (mainly pulmonary) in some studies, but not all [3•, 5, 19, 20]. Disseminated forms and cerebral mucormycosis are associated with the worst outcome with up to 80% mortality rates [3•, 20].

Considering the global increasing incidence of IM and its particularly high mortality rate, there is an urgent need to improve our diagnostic and therapeutic approaches of this fungal disease. The aim of this review is to present the limitations of the current “state of the art” in the management of mucormycosis and to discuss research developments and novel perspectives for the future, which are summarized in Table 1.

**Table 1:** Management of invasive mucormycosis: present and future

Current options	Limitations	Perspectives
Diagnosis		
Direct exam, culture, PCR	Low sensitivity of culture Lack of standardization and limited availability of PCR Lack of a non-invasive test for specific biomarker detection	Development of LFA for Mucorales-specific antigen detection (e.g. α-1,6 mannan, extracellular polysaccharide) in serum or BAL Development of multiplex PCR kits Immuno-PET approaches
Therapy		
Antifungal drugs Amphotericin B lipid formulations and triazoles (posaconazole, isavuconazole)	Drug toxicity and DDI Limited options for oral therapy	Modified molecules from existing antifungal drug classes with less toxicity/DDI and/or oral bioavailability (e.g. tetrazoles, encochleated amphotericin B) Novel antifungal drug classes with distinct mechanism of action (e.g. fosmanogepix) Specific antibodies (anti-CotH or anti-integrin β1 antibodies)
Adjunctive measures Surgery Hyperbaric oxygen Iron chelators Correction of immunosuppression whenever possible Correction of hyperglycemia	Limited or no demonstrated efficacy except for surgery	Strategies to enhance the immune response (interferon-γ, nivolumab, CAR-T cells)

*PCR*, polymerase chain reaction; *LFA*, lateral-flow assays; *BAL*, bronchoalveolar lavage; *PET*, positron emission tomography; *DDI*, drug-drug interactions; *CAR*, chimeric antigen receptor

## Diagnosis of Mucormycosis

### Current Approach

While the clinical presentation of ROC mucormycosis is very suggestive of the diagnosis in a patient at risk, pulmonary mucormycosis cannot be distinguished from other invasive mold infections, unless a reverse halo sign is present at chest CT, which is a good predictor of IM [21, 22]. Extrapulmonary IM in immunocompromised hosts (e.g. abdominal, cerebral) is not associated with any specific clinical or radiological presentation and may be misdiagnosed.

The microbiological diagnosis of IM is particularly difficult because of the low yield of cultures from clinical samples with 25 to 40% of cases being documented by histopathology only (i.e. presence of broad non-septate hyphae suggestive of Mucorales) [3•, 20, 23••]. The diagnosis of IM usually requires invasive procedures for the acquisition of deep tissue samples, which may result in significant delays. Rapid non-invasive diagnostic tools are needed for early diagnosis and prompt initiation of targeted antifungal therapy. The current commercialized kits for the detection of fungal biomarkers, such as the galactomannan and 1,3-β-d-glucan, cannot detect IM [24•]. Mucorales-specific PCRs currently represent the most sensitive approach and the unique diagnostic test that can be performed in serum for early diagnosis of IM. However, there is a lack of commercial and standardized methods [25••]. A combination of three real-time PCR targeting *Mucor*/*Rhizopus*, *Rhizomucor* and *Lichtheimia* spp. showed a sensitivity of 80–85% for detection of probable or proven IM in serum samples, which preceded diagnosis by conventional methods [26••, 27, 28]. This multiplex PCR also showed good sensitivity and specificity (100% and 97%, respectively) for the diagnosis of IM in bronchoalveolar lavage (BAL) samples [29]. Recently, a multiplex real-time PCR kit for a large panel of pathogenic Mucorales has been marketed (MucorGenius™, Pathonostics, Maastricht, The Netherlands) and demonstrated similar sensitivity for IM detection in serum samples (75%) and in pulmonary samples (100%) [30, 31]. Because of the low sensitivity of culture in tissue samples, use of in-house pan-Mucorales PCR assays targeting the conserved ribosomal DNA (mainly 18S rDNA or 28S rDNA and internal transcribed spacer [ITS] region) are helpful for diagnosis in native or formalin-fixed paraffin-embedded (FFPE) tissue samples [32–34].

### Future Directions

The lack of a Mucorales-specific fungal biomarker that can be easily detected in serum is still a gap in the diagnostic approach of IM. Indeed, a test for such biomarkers may be cheaper and more broadly available than PCR. While Mucorales do not produce galactomannan and have few 1,3-β-d-glucan, other polysaccharides of their cell wall could be used as targets. A lateral flow immunoassay (LFA) with an antibody binding to α-1,6 mannan has been developed and showed good in silico performance for the detection of Mucorales (*Mucor* and *Rhizopus*) from culture extracts [35]. A clinical evaluation is still lacking. Another LFA targeting an extracellular polysaccharide, which is specific to *Rhizopus arrhizus*, has been developed and was successfully tested in spiked human serum and BAL [36].

Novel PCR approaches use targets that are more sensitive or more specific for discrimination of Mucorales at species level compared to the ribosomal DNA targets. PCR targeting the mitochondrial DNA (e.g. *rnl* gene, encoding for large-subunit ribosomal RNA) was more sensitive compared to PCR targeting nuclear ribosomal RNA (18S rDNA) for detection of Mucorales in FFPE tissue samples and more accurate for species identification [37]. Another candidate for a more specific detection of Mucorales is the spore coat protein (CotH), which is essential for angio-invasion via binding to the glucose-regulated protein 78 (GRP78) at the surface of endothelial cell [38, 39]. A PCR assay for CotH detection in urine demonstrated good performance in a murine model of IM and in patients with proven IM (about 90% sensitivity and 100% specificity) [40].

Metagenomic next generation sequencing has also demonstrated some utility for the early diagnosis of IM in case reports when other conventional methods have failed [41–43].

A preliminary study analyzing murine and human breath samples by thermal desorption gas chromatography/tandem mass spectrometry (GC–MS/MS) found distinct volatile metabolite (sequiterpene) profiles for the most frequent pathogenic Mucorales [44]. Such non-invasive approach might deserve further investigation as a diagnostic tool for IM or a screening strategy in high-risk patients.

Novel perspectives for the diagnosis of IM should also consider the use of positron emission tomography (PET) and magnetic resonance imaging (MRI) with a fungal specific radiolabeled marker, such as monoclonal antibodies or iron siderophores. These approaches, which have been studied in animal models for the diagnosis of invasive aspergillosis [45–50], have not yet been investigated for IM diagnosis.

## Treatment of Mucormycosis

### Current Approach

The treatment of IM relies on three major axes of equal importance: (i) early appropriate antifungal therapy, (ii) source control by surgery, and (iii) correction of immunosuppression [25••].

Only two antifungal drug classes are approved for the treatment of IM, the polyene amphotericin B and the new generation triazoles posaconazole and isavuconazole [25••]. Liposomal amphotericin B (L-AmB) at high doses (5–10 mg/kg/day) is the preferred first-line treatment. Its use is limited by renal toxicity and the lack of an oral formulation. In retrospective analyses of large cohorts, L-AmB was associated with lower mortality rates compared to that observed with other antifungal regimens [3•, 51, 52]. Posaconazole or isavuconazole, which are available as intravenous or oral formulations, are alternative options in case of preexisting or acute renal insufficiency and are privileged for maintenance outpatient therapy [25••]. In a single arm open-label trial, isavuconazole as first-line treatment of IM demonstrated similar success rates compared to those observed in a historical cohort of patients treated with amphotericin B formulations [23••]. Although posaconazole salvage therapy has been associated with acceptable success rates, data of its efficacy as first-line treatment of IM are lacking [53, 54]. It is noteworthy that the in vitro activity of posaconazole and isavuconazole against the Mucorales exhibits inter- and intra-species/genus variability with some isolates (mainly *Mucor circinelloides* and *Rhizopus* spp.) exhibiting high minimal inhibitory concentrations (MICs) [55], but there is no evidence that these differences affect the outcome [23••, 53, 54]. Combination therapies of L-AmB with either an echinocandin (caspofungin) or posaconazole are marginally recommended. The adjunction of caspofungin or anidulafungin to L-AmB or amphotericin B lipid complex showed improved efficacy compared to the monotherapies in a murine model of IM [56, 57]. However, results of clinical studies assessing the potential benefit of this combination were controversial [18, 19, 58, 59]. The combination of posaconazole and L-AmB did not demonstrate any synergism in a murine model of IM and there is no evidence of its clinical benefit, although its use has been reported [60, 61]. In the absence of comparative prospective studies, the actual impact of antifungal drug combinations on outcome cannot be assessed because of multiple biases. Indeed, many clinicians would favor combined therapies for more severe or refractory cases.

There is strong evidence that antifungal therapy alone is not sufficient to cure mucormycosis and that surgical source control should be performed whenever possible. Surgery was associated with a higher survival rate in several cohort studies of IM [2, 3•, 5, 19, 20, 52, 62–64]. Surgery should be early and as complete as possible, which may be difficult to achieve in the setting of profound thrombopenia and neutropenia in hematologic cancer patients, and in case of multiple infectious foci or invasion of adjacent anatomical structures.

Recovery of neutropenia is also a predictor of better outcome in hematologic cancer patients [62]. Recombinant human granulocyte–macrophage or granulocyte colony-stimulating factor (GM-CSF or G-CSF) is frequently used to accelerate neutrophil recovery following myeloablative chemotherapy of hematologic cancer, and their use as adjunctive therapy for the treatment of invasive mold infections has been considered [65]. In a murine model of *Rhizopus oryzae* disseminated infection, the adjunction of GM-CSF to L-AmB therapy could prolong survival and reduce fungal burden compared to L-AmB treatment alone [66]. Some case-series, especially in the pediatric population, reported promising results of adjunctive GM-CSF therapy for refractory IM, but comparative studies are lacking [67, 68]. Use of granulocyte transfusion as secondary prophylaxis or adjunctive treatment of IM or other invasive fungal infections has also been reported [69, 70]. However, one retrospective study showed that patients with invasive pulmonary aspergillosis who received adjunctive granulocyte transfusion had a high rate of pulmonary reactions and an overall worse outcome compared to those who were treated by antifungal therapy alone [71]. In patients receiving corticosteroid therapy, tapering of corticosteroid dosing is recommended whenever possible [25••]. In diabetic patients with IM, control of the underlying conditions with reversion of hyperglycemia and ketoacidosis is also warranted [25••].

Hyperoxic and hyperbaric conditions have an inhibitory effect on the growth of Mucorales in vitro and may also enhance the antifungal activity of amphotericin B and favor tissue healing [72, 73]. This practice has been occasionally applied for the treatment of IM with an overall good survival rate in case reports or case-series [2, 73]. One retrospective cohort study of hematologic cancer patients with IM found that treatment with hyperbaric oxygen was associated with better outcome [19]. However, prospective randomized comparative studies are lacking to demonstrate its benefit and this approach received a moderate strength of recommendation in guidelines [25••].

Because iron homeostasis is important for the virulence of *Mucorales*, iron chelators have been considered as adjunctive therapy of IM. Deferasirox showed promising results with synergistic effect in combination with L-AmB for the treatment of IM in diabetic mice [74]. In neutropenic mice, deferasirox was effective only when administered with a prolonged interdose interval, although no evidence of toxicity could be demonstrated [74]. Although deferasirox was safe for the treatment of IM in a small open-label case series or as compassionate use [75, 76], it failed to demonstrate its efficacy in combination with L-AmB and was even associated with a worse outcome compared to L-AmB monotherapy in a small placebo-controlled trial [77]. These results could be explained by an imbalance between both groups with a predominance of neutropenic patients in the deferasirox arm. While iron chelators cannot be recommended for the treatment of IM on the basis of current data, their utility in diabetic patients should be further investigated [25••].

### Future Directions

Novel antifungal drugs are needed for the treatment of IM because of the limited efficacy and the potential toxicity of the only two available antifungal drug classes (i.e. amphotericin B and triazoles).

Novel antifungal drugs that are currently in phase II/III clinical trials include some first-in-class molecules (fosmanogepix, olorofim, ibrexafungerp) and modified compounds from existing antifungal drug classes with improved pharmacologic properties (rezafungin, tetrazoles, encochleated amphotericin B) [78].

Among the first-in-class antifungals, only fosmanogepix displays some effect against Mucorales. Fosmanogepix is an inhibitor of the glycosylphosphatidylinositol (GPI) biosynthesis pathway, which is required for the anchorage of mannoproteins in the cell membrane and cell wall [79]. It has a potent fungistatic effect with broad spectrum activity against most pathogenic yeasts and molds [78]. However, it has limited in vitro activity against Mucorales with MIC_50_ (i.e. encompassing 50% of isolates) of 4 to 16 mg/L [80]. The efficacy of fosmanogepix was tested in mice infected by two strains of *Rhizopus arrhizus* with MICs of 0.25 and 4 mg/L, respectively [81]. Fosmanogepix demonstrated a significant effect against both strains versus the placebo, which was comparable to that of isavuconazole. In another murine experiment using a *R. arrhizus* strain with low fosmanogepix MIC (0.25 mg/L), fosmanogepix was equally effective than L-AmB and the combination of both drugs was synergistic [82]. Although these results are encouraging, data about the efficacy of fosmanogepix against other species of Mucorales including isolates with higher MICs are needed. A small phase II non-comparative open-label study assessing the efficacy of fosmanogepix for the treatment of invasive aspergillosis and other invasive mold infections including IM has just been completed and results are expected (AEGIS, NCT04240886). While fosmanogepix displays some interesting properties, such as its oral bioavailability and its good penetration in the central nervous system, its potential for a future application in the treatment of IM seems limited.

The tetrazoles (VT-1161, VT-1598) have similar mechanism of action than other azole compounds, but a decreased affinity for human cytochrome P450 isoenzymes, which results in less hepatotoxicity and drug-drug interactions [83]. VT-1598 and VT-1161 displayed variable activity against *R. arrhizus* var. *arrhizus*, but not var. *delemar* [84, 85]. VT-1161 was effective in preventing and treating mice infected with *R. arrhizus* var. *arrhizus* [84, 86]. Data about the in vitro and in vivo efficacy of tetrazoles against other Mucorales are lacking.

To overcome the lack of oral bioavailability and toxicity of amphotericin B, a novel formulation of encochleated drug has been developed. Cochleates consist of a calcium-phospholipid anhydrous crystal that prevents hydrophobic amphotericin B from degradation in the gastro-intestinal tract and enables targeted delivery of the drug in the macrophages with reduced toxicity [87••]. In a phase I study, oral encochleated amphotericin B was well tolerated with only minor adverse events and no renal toxicity [88••]. Two phase II studies have assessed its efficacy for the treatment of chronic mucocutaneous and vulvovaginal candidiasis [87••]. Pre-clinical and clinical data of the efficacy of oral encochleated amphotericin B for the treatment of IM are currently lacking.

Among the novel antifungal agents that are in pre-clinical stage of development, strategies targeting the spore coat proteins homologs (CotH), which play a specific role in the virulence of the Mucorales, may represent the most promising perspective for the treatment of IM. CotH are a family of kinase proteins playing a role in morphogenesis (e.g. spore formation, cell wall structure), stress adaptation and virulence [89•]. CotH3, a surface protein of the Mucorales, binds to the glucose-regulated protein 78 (GRP78) receptor at the surface of human nasal epithelial cells and endothelial cells, which enables tissue invasion and angio-invasion [38, 39, 90•]. Elevated concentrations of iron, hyperglycemia and ketone bodies increase the expression of the GRP78 receptor and anti-GRP78 antibodies were shown to protect diabetic ketoacidotic (DKA) mice from IM [39]. Conversely, a *Rhizopus oryzae* mutant with reduced CotH3 expression exhibited a loss of ability to invade endothelial cells and decreased virulence in a DKA murine model of IM [38]. A similar effect could be obtained by polyclonal antibodies against CotH3, which was shown to protect both DKA and neutropenic mice from IM [91••]. This mechanism of pathogenicity seems to be predominant in patients with uncontrolled diabetes who are more prone to develop ROC mucormycosis. In pulmonary mucormycosis, which affects mainly hematologic cancer patients, lung invasion and angio-invasion seems to be mediated by another CotH protein (CotH7) via the integrin β1 receptor expressed at the surface of alveolar epithelial cells and subsequent activation of the epidermal growth factor receptor (EGFR) [90•]. Indeed, anti-integrin β1 antibodies could protect mice from pulmonary IM [90•]. These distinct pathways of pathogenesis may explain the predisposition of diabetic patients to develop ROC mucormycosis. The GRP78 receptor was also shown to play a role in recognition of the SARS-CoV-2 spike protein and to enable the translocation to the lung epithelial cell membrane of the angiotensin–converting enzyme 2 (ACE2) receptor, the major ligand of the virus [92]. The stress resulting from severe COVID-19 may therefore result in overexpression of GRP78 and favor IM [93]. This better understanding of the pathogenesis of IM opens perspectives for individualized therapeutic approach according to the type of underlying conditions and localization of the disease.

Because restoration of the host immune is another crucial determinant for the outcome of IM, strategies to boost the immune system are also investigated. Adjunctive therapy with interferon-γ and/or the checkpoint inhibitor anti-PDR1 antibody nivolumab has been used with success to treat refractory IM in some case reports [94–96]. This approach would deserve further investigation in randomized controlled trials.

Other strategies would consist of generating a specific and targeted response against the fungal pathogen. Ex vivo genetically modified T cells expressing a fungal-specific chimeric antigen receptor (CAR), such as dectin-1, demonstrated in vitro and in vivo (murine model) effect against *Aspergillus* [97]. Transfusion of leucocytes loaded ex vivo by the antifungal drug posaconazole could enhance activity of the drug in a murine model of invasive aspergillosis [98]. Such approaches have not yet been tested for the treatment of IM.

## Conclusion

Despite improved diagnostic approaches with the development of molecular methods (e.g. PCR) and novel therapies (e.g. isavuconazole), the prognosis of IM remains poor with no significant decrease of the mortality rates over the last decade. The recent development of international guidelines for the management of IM and the development of a tool, the European QUALity (EQUAL) score, to assess adherence to these guidelines, may be helpful to improve the standardization of the management and the prognosis of IM [25••, 99, 100].

Because of the extremely aggressive and invasive course of the disease, the management of IM should involve multiple and distinct approaches, including screening for early detection, prompt appropriate antifungal therapy, surgery, correction of the underlying predisposing conditions (i.e. immunosuppression or hyperglycemia) and strategies to boost the immune response. While novel antifungal drugs that are currently in phase II/III clinical trials, such as fosmonagepix, olorofim or ibrexafungerp, seem to have no or little place for the treatment of IM, other antifungal strategies, which are currently at a pre-clinical stage, such as CotH3 or anti-integrin β1 antibodies, look promising. Strategies focusing on the host immune response, such as interferon-γ or anti-PDR1 antibody (nivolumab) also deserve further investigation in randomized controlled trials. Because IM is a relatively rare disease, it is difficult to initiate such large clinical trials and to get support from funding agencies for laboratory and clinical research. However, the recent burden of IM in the setting of the COVID-19 pandemic has brought more attention to this severe disease.
